# Molecular identification and characterization of *Trichinella spiralis* proteasome subunit beta type-7

**DOI:** 10.1186/s13071-014-0626-z

**Published:** 2015-01-13

**Authors:** Wei Yang, Ling Ge Li, Ruo Dan Liu, Ge Ge Sun, Chun Ying Liu, Shuai Bing Zhang, Peng Jiang, Xi Zhang, Hui Jun Ren, Zhong Quan Wang, Jing Cui

**Affiliations:** Department of Parasitology, Medical College of Zhengzhou University, Zhengzhou, 450052 P. R. China; Department of Laboratorial Medicine, The First Affiliated Hospital of Zhengzhou University, Zhengzhou, 450052 P. R. China

**Keywords:** *Trichinella spiralis*, Proteasome subunit beta type-7 (Tspst), Invasion, Immune protection

## Abstract

**Background:**

Previous study showed that *Trichinella spiralis* proteasome subunit beta type-7 (Tspst) gene is an up-regulated gene in intestinal infective larvae (IIL) compared to muscle larvae (ML), which was screened by using suppression subtractive hybridization (SSH) and confirmed by real-time PCR. Tspst may be related to the larval invasion of intestinal epithelial cells (IECs). The aim of this study was to identify Tspst and to investigate its immune protection against intestinal *T. spiralis* infection.

**Methods:**

The Tspst gene encoding a 29 kDa protein from *T. spiralis* infective larvae was cloned, and recombinant Tspst protein (rTspst) was produced in an *Escherichia coli* expression system. The rTspst was used to immunize BALB/c mice. Anti-rTspst antibodies were used to determine the immunolocolization of Tspst in the parasite. Transcription and expression of Tspst at *T. spiralis* different developmental stages were observed by RT-PCR and immunofluorescence test (IFT). The *in vitro* or *in vivo* immune protection of anti-rTspst serum or rTspst against intestinal *T. spiralis* infection in BALB/c mice was evaluated.

**Results:**

Anti-rTspst serum recognized the native Tspst protein with 29 kDa in ML crude antigens. Transcription and expression of gene was observed at all *T. spiralis* different developmental stages (IIL, adult worms, newborn larvae, and ML). An immunolocalization analysis identified Tspst in the cuticle and internal organs of the parasite. An *in vitro* invasion assay showed that, when anti-rTspst serum, serum of mice infected with *T. spiralis* or normal mouse serum were added to the medium, the invasion rate of the infective larvae in an IEC monolayer was 25.2%, 11.4%, and 79%, respectively (*P* < 0.05), indicating that anti-rTspst serum partially prevented the larval invasion of IECs. After a challenge infection with *T. spiralis* muscle larvae, mice immunized with rTspst conferred a 45.7% reduction in adult worm burden in intestines.

**Conclusions:**

In the present study, Tspst was first identified and characterized. Tspst is an invasion-related protein of *T. spiralis* IIL and could be considered as a potential vaccine candidate antigen against intestinal *T. spiralis* infection that merits further study.

## Background

*Trichinella spiralis* is a tissue-dwelling parasitic nematode that infects humans and other mammals. Infection occurs by the consumption of raw or undercooked meat from infected animals (e.g., pigs and wild animals) [1,2]. Once ingested, muscle larvae (ML) are released from their capsules in the stomach by the digestive enzymes, and activated into the intestinal infective larvae (IIL) by intestinal contents or bile after 0.9 hour post-infection (hpi), and interacted with host intestinal epithelial cells (IECs) [3,4]. Then, the IIL penetrate into host intestinal epithelium where they molt four times during 10–28 hpi, and mature into adults that mate and produce the next generation of larvae [5,6]. So, the invasion of host IECs by the infective larvae is the first step during *T. spiralis* infection.

*T. spiralis* larvae do not possess oral appendices or a spike [7], implying that the larval invasion of IECs may be not simply a result of mechanical penetration but possibly mediated by excretory-secretory (ES) proteins produced by the infective larvae [8,9]. Moreover, after the ML were activated by bile, the activated-larvae produce or excrete a few proteins [10]. When the *T. spiralis* infective larvae were inoculated onto the monolayers of IECs, they invade the IECs and produced several proteins, and some of these proteins entered the IECs [11,12]. These proteins might be related with the larval invasion of IECs. However, the biological functions of the larval invasion-related proteins and the molecular mechanism of protein-IEC interaction are unknown.

Our previous studies showed that some genes of *T. spiralis* are differentially expressed between ML and IIL [13]. *T. spiralis* proteasome subunit beta type-7 (Tspst) gene was an up-regulated gene in IIL compared with that of ML, which was identified by using suppression subtractive hybridization (SSH) and then confirmed by real-time PCR [14]. The Tspst might be related with the larval invasion of IECs, but their exact biological functions are unknown. The proteasome is highly conserved during eukaryotic evolution. It is an essential component of the ATP-dependent proteolytic pathway in eukaryotic cells and is responsible for the degradation of most cellular proteins. Proteasome-mediated proteolysis refers to a selective cellular process that results in the specific degradation of either run-needed (but normal) or damaged/misfolded proteins. Proteasome is a major mechanism by which cells regulate the concentration of particular proteins and degrade misfolded proteins [15,16]. In parasites, the proteasome is involved in the regulation of cell differentiation and replication, participation in impairment on hosts, and could be an antigen inducing immune response in host [17,18]. Studies on parasite proteasomes will be beneficial for better understanding of mechanisms in the invasion of host cell by parasites, also providing a new insight for prevention and control of parasitic diseases. However, to the best of our knowledge, there has been no report of the characterization and functional analysis of the Tspst.

In the present study, the Tspst gene (GenBank accession No. XM_003374391.1) encoding a 29 kDa protein from *T. spiralis* infective larvae was cloned and identified. The expression, immunolocalization of Tspst and the immune protection of the recombinant Tspst protein (rTspst) against the intestinal *T. spiralis* infection in mice was also investigated.

## Methods

### Parasites and experimental animals

The isolate (ISS534) of *T. spiralis* used in this study was obtained from domestic pigs in Nanyang, Henan Province, China. The isolate was maintained by serial passage in BALB/c mice every 6–8 months. Specific pathogen-free (SPF) female BALB/c mice aged 6 weeks were purchased from the Experimental Animal Center of Henan Province and used for the immunological studies and challenge infection. All the animal experiments reported herein were approved by The Life Science Ethics Committee of Zhengzhou University.

### Collection of worms and preparation of crude and ES antigens

*T. spiralis* ML from infected mice at 42 days post-infection (dpi) were recovered by digestion of carcasses with 0.33% pepsin (1:31000; Sigma) and 1% HCl [19]. Adult worms (AW) were isolated from the small intestines of infected mice at 3 and 7 dpi [20]. The newborn larvae (NBL) were collected from female adult worms cultured in RPMI-1640 medium containing 10% fetal bovine serum (FBS; Gibco) in 5% CO_2_ at 37°C for 24 h [21].

The ES antigens of the ML were prepared as previously described [22]. In brief, after washing thoroughly in sterile saline, the larvae were again washed four times in serum-free RPMI-1640 medium supplemented with 100U/ml penicillin and 100U/ml streptomycin. The larvae were incubated in the same medium at concentration of 5 000 worms/ml for 18 h at 37°C in 5% CO_2_. After incubation, the media contained the ES products were filtered through a 0.2 μm membrane into a 50-ml conical tube, then centrifuged at 4°C, 15,000 × g for 30 min. The supernatant was dialyzed against deionized water at 4°C for 2 days, and then concentrated by a vacuum concentration and freeze drying (Heto Mxi-Dry-Lyo, Denmark), respectively.

The crude (somatic) antigens were prepared from *T. spiralis* ML resuspended in deionized water. The suspension was submitted to 5 cycles of freezing-thawing. The larvae were homogenized on ice in a glass tissue grinder. After this, the larval fragments were further homogenized with ultrasonication (99 times 3-s cycle, 100 W, 0°C). The supernatant was collected after centrifugation at 15,000 g for 1 h at 4°C [23].

The protein concentration of ES antigens (1.09 mg/ml) and crude antigens (1.25 mg/ml) was determined by the method described by Bradford [24].

### Cell culture

In our experiments, normal mouse IECs used were obtained from fetal mouse small intestines and were susceptible to invasion by *T. spiralis* [25]. The IECs were cultured (5% CO_2_, 37°C) in complete DMEM containing 4 mM glutamine, 20 mM Hepes, 1 mM sodium pyruvate, 100 U/ml penicillin, 100 U/ml streptomycin, 0.1 U/ml bovine insulin (Sigma), and 10% fetal bovine serum (Gibco). The cells were used at passage 9 for the experiment. Cell monolayer was dispersed by trypsinization (0.5% trypsin–0.54 mM EDTA in PBS, at 23°C for 5 min).

### Cloning, expression, and identification of Tspst

Total RNA was extracted from the ML using Trizol (Invitrogen). The first-strand synthesis of cDNA was accomplished using AMV reverse transcriptase (Promega, USA) and oligo (dT) primers at 42°C for 1 h according to the manufacturer’s instructions. The Tspst gene was amplified by PCR, and specific primers carrying *Bam*HI and *Hind*III restriction enzyme sites (Forward, 5′-CATGGATCCATGGAAGACGCTATTATATCATCTG-3′; Reverse, 5′-CATAA GCTTTCATTCCATCATTTTAGTGCTTGAAAC-3′) were used. The cycling protocol was as follows: 35 cycles of 94°C for 45 s, 56°C for 45 s and 72°C for 1 min. The purified PCR products were cloned into the expression vector pMAL-C2X (Novagen, USA) using the *Bam*HI and *HindIII* sites. The recombinant plasmid was then transformed into *Escherichia coli* BL21 (Novagen, USA). The expression of the recombinant protein was induced with 0.5 mM IPTG at 37°C for 4 h. The pellets of the bacterial culture were harvested following the induced incubation and disrupted by sonication in 20 mM Tris–HCl/0.2 mM NaCl buffer (pH 7.4), and the recombinant proteins was expressed in supernatant. The rTspst was purified by Amylose Pre-packed Column (NEB Ltd, China). The purified rTspst was analyzed by sodium dodecyl sulfate-polyacrylamide gel electrophoresis (SDS–PAGE) using a 5% acrylamide stacking gel and 12% acrylamide separating gel (83 × 73 × 1.0 mm) with a Mini-PROTEAN 3 Cell electrophoresis unit (BioRad, USA) at 120 V for 2.5 h [26]. After electrophoresis, the gel was stained with 0.25% Coomassie brilliant blue R-250 for 4 h and then destained (10% acetic acid and 5% ethanol). Another gel was prepared in the same way and used for the Western blot analysis described below.

### Generation of anti-rTspst antibodies

Pre-immune sera of ten female BALB/c were collected by tail bleeding 2 days prior to the first immunization. The BALB/c mice were subcutaneously immunized with 20 μg of rTspst emulsified with complete Freund’s adjuvant (CFA), followed by three boosts with the same amount of protein emulsified with incomplete Freund’s adjuvant at 10-day intervals. Seven days after the last boost, the mice were bled, and the sera were collected.

### Antibody determination

The specific IgG antibodies to rTspst in serum samples of immunized mice were determined by ELISA using corresponding rTspst protein, ES or crude antigens. The procedure of ELISA was performed as previously described [23]. Briefly, microtiter plates (Nunc) were coated with 2.5 μg/ml of rTspst proteins, ES or crude antigens in coating buffer overnight at 4°C, and blocked with 200 μl of PBS-0.1% Tween 20 (PBST) containing 5% skimmed milk. Then, 100 μl of immune serum with 1:100 dilutions in PBS were added to each well and incubated at 37°C for 1 h. HRP-conjugated goat anti-mouse IgG antibodies (1:5000; Southern Biotechnology, USA) were added and incubated at 37°C for 1 h. The plates were developed with o-phenylenediamine dihydrochloride substrate (OPD; Sigma), and the absorbance was measured at 490 nm.

### Western blot analysis

Samples including crude antigens (15 μg/lane), ES antigens (15 μg/lane) and the rTspst proteins (3 μg/lane) were separated by SDS–PAGE and then transferred onto nitrocellulose membranes (Millipore, USA) using a trans-blot SD transfer cell (Bio-Rad, USA) [27]. The membranes were cut into strips, blocked with 5% skimmed milk in Tris-Buffered Saline with Tween-20 (TBST) at 37°C for 1 h, and incubated at 37°C for 1 h with 1:100 dilutions of different mouse sera (anti-rTspst serum, serum from mice infected *T. spiralis* at 30 dpi and normal mouse serum). After washing, the strips were incubated at 37°C for 1 h with HRP-conjugated goat anti-mouse IgG (1:5000 dilution; Southern Biotechnology, USA), and finally with 3, 30-diaminobenzidine tetrahydrochloride (DAB; Sigma).

### RT-PCR analysis of Tspst gene transcription

To observe the transcription of the Tspst gene at different developmental stages of *T. spiralis*, total RNA was extracted from the IIL, Ad, NBL and ML of *T. spiralis*. RT-PCR was performed as previously described [28]. The housekeeping gene GAPDH (glyceraldehyde-3-phosphate dehydrogenase, GenBank accession No. AF452239) of *Trichinella* was used as a constitutively expressed standard gene, the primers were designed as follows: forward, 5′-TTAATGTCGTGGCTGTGAAT-3′, and reverse, 5′-CCAGTAG AAGCAGGGATGAT-3′.

### Immunofluorescence test (IFT)

IFT was used to observe the expression of Tspst gene at different developmental stages and its immunolocalization in the parasite. The intestines (at 2 hours post infection, 3 and 7 dpi) and skeletal muscles (at 19 and 42 dpi) from mice infected with *T. spiralis* were collected respectively, and were fixed in 4% paraformaldehyde and embedded in paraffin. Microtome-cut 2-μm sections were placed on slides, deparaffinized in xylene and rehydrated. The whole parasites and tissue sections were blocked with 5% normal goat serum in PBS and then incubated in a moist chamber at 37°C for 1 h with a 1:10 dilution of immune sera, infection sera or normal sera. After being washed three times in PBS, the whole parasites and sections were incubated with a 1:50 dilution of FITC-labeled goat anti-mouse IgG (Santa Cruz, USA), washed five times in PBS, and examined under a fluorescent microscope (Olympus, Japan) [9].

### Invasion assay

ML were activated by the mouse bile (diluted 1:20 in saline) at 37°C in 5% CO_2_ for 2 h [8]. The bile was removed prior to analysis by exhaustively washing the worms in PBS supplemented with 100 U/ml penicillin and 100 U/ml streptomycin, and the larvae were incubated in PBS at 37°C in 5% CO_2_ for additional 1 h [29]. When the IECs cells were grown to confluence in 6-well plates (Corning, USA), each monolayer was overlaid with approximately 150 bile-activated larvae suspended in 2 ml of semisolid medium (serum-free DMEM containing 15 mM HEPES and 1.75% agarose) mixed with a 1:10 dilution of anti-rTspst serum, serum from mice infected with *T. spiralis*, normal mouse serum [8]. After incubation at 37°C for 1 h, the partial invasion of IECs by the infective larvae was observed using inverted phase-contrast microscope (Olympus, Japan), and the number of larvae in the cell monolayer was counted [25].

### Immune protection against challenge infection

To determine the immune protection of the rTspst, a total of 40 female BALB/c mice were divided into four groups (immune group, maltose-binding protein (MBP) tag control group, adjuvant control group and PBS control group) of 10 mice each. The immune groups of BALB/c mice were subcutaneously immunized with 20 μg of the purified rTspst protein. The vaccines were prepared at a 1:1 ratio by mixing rTspst protein in complete Freund’s adjuvant (CFA) for the first immunization or incomplete Freund’s adjuvant for the subsequent immunizations (three boosts at 10 day-intervals) and administered intradermally at multiple sites of the abdomen. The control group received PBS with the corresponding adjuvant, MBP tag or PBS only. Ten days after the last immunization, all the four groups of mice were orally challenged with 300 *T. spiralis* muscle larvae. Ten mice from each group were euthanized 7 days after challenge and the numbers of intestinal adult worms were counted [30,31]. The intestinal protective immunity was calculated as the worm reduction rate of recovered adult worms in intestine from the immunized group versus those from the control groups [26].

### Statistical analysis

All of the statistical analyses of the data were performed using SPSS for Windows, version 17.0 (SPSS Inc., Chicago, IL). The AW recovery data were expressed as the mean value ± standard deviation, and the differences among the groups were analyzed using the one-way ANOVA method. The statistical significance was defined as *P* < 0.05.

## Results

### Molecular cloning and expression of a cDNA encoding Tspst

**Figure 1:**
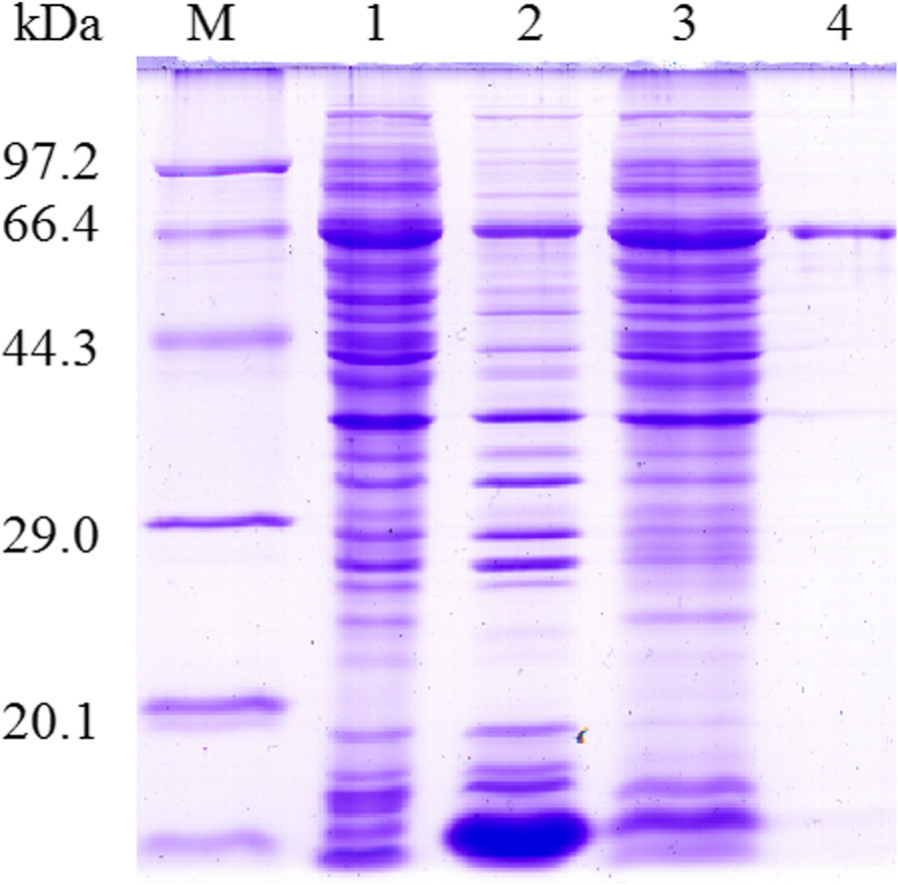
**The SDS-PAGE analysis of recombinat Tspst protein purified by Amylose Pre-packed Column.** M: protein molecular weight marker; 1: the lysis of the induced recombinant bacteria after ultrasonication; 2: recombinant Tspst proteins (rTspst) was expressed in precipitation; 3: rTspst was expressed in supernatant; 4: rTspst purified by Amylose Pre-packed Column.

The full coding sequence of Tspst gene was cloned into the prokaryotic expression plasmid pMAL-C2X. After being induced with 0.5 mM IPTG, BL21 bacteria harboring pMAL-C2X-Tspst expressed a 72 kDa fusion protein. On SDS-PAGE analysis, the molecular size of the rTspst was consistent with the predicted combined size of the protein encoded by the cDNA clone (29 kDa) and MBP tag from the vector (43 kDa) (Figure 1).

### Western blot and ELISA analysis of the recombinant Tspst protein

**Figure 2:**
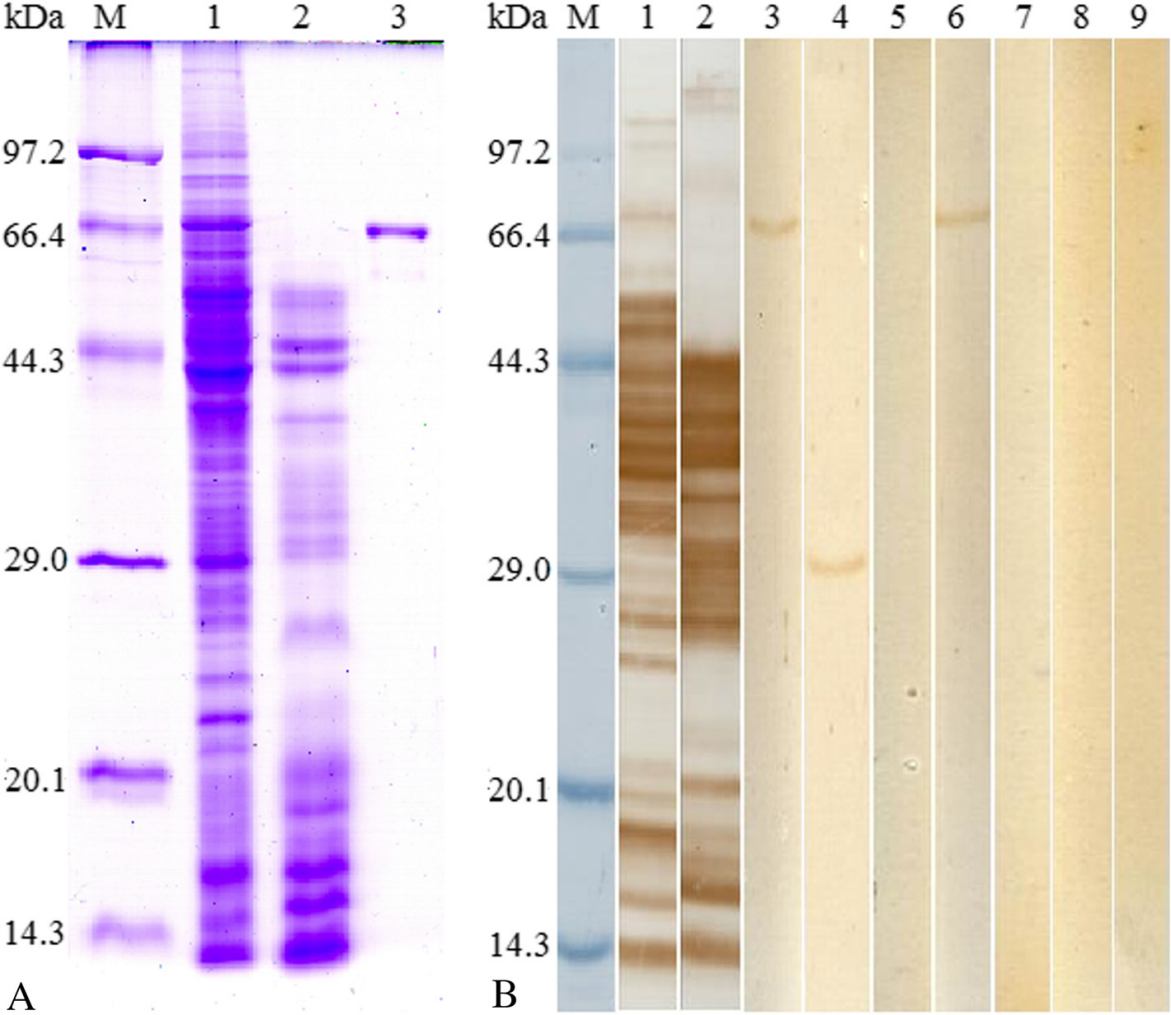
**Identification of recombinant Tspst protein. A**. SDS-PAGE analysis of recombinant Tspst protein (rTspst). M: protein molecular weight marker; 1: *T. spiralis* ML crude antigens (15 μg/lane); 2: *T. spiralis* ML ES antigens (15 μg/lane); 3: rTspst (3 μg/lane) purified by Amylose Pre-packed Column. **B**. Western blot analysis of rTspst antigenicity. The *T. spiralis* ML crude antigens (1), ES antigens (2), and rTspst (3) were recognized by sera of mice infected with *T. spiralis* at 30 dpi*.* The native Tspst protein in crude antigens (4) and rTspst (6) were recognized by anti-rTspst serum, but the ES antigens (5) were not recognized by anti-rTspst serum. The *T. spiralis* crude antigens (7), ES antigens (8), and rTspst (9) were not recognized by normal mouse sera.

**Figure 3:**
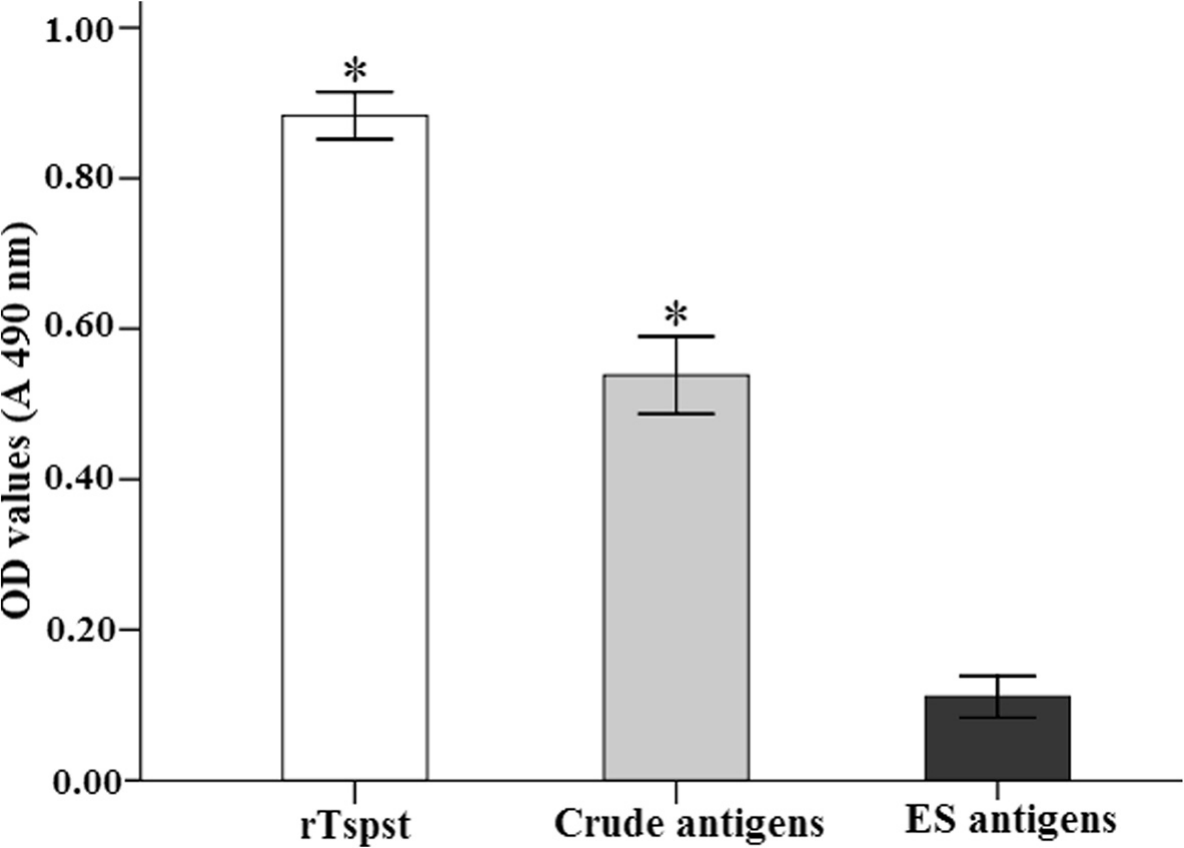
**The specific IgG antibody levels of mice immunized with rTspst assayed by ELISA using different antigens.** The optical density (OD) values shown for each group are the mean ± standard deviation (SD) of antibody levels (n = 10). Asterisks (*) indicate statistically significant differences (P < 0.01) in OD values of ES antigens compared to rTspst or crude antigens.

Western blot analysis showed that the rTspst was recognized by an anti-rTspst serum and serum from mice infected *T. spiralis* at 30 dpi. The 29 kDa protein components of the crude antigens of *T. spiralis* ML were recognized by the anti-rTspst serum (Figure 2). The results of ELISA showed that anti-rTspst serum recognized the rTspst and crude antigens, but did not recognize the ES antigens (Figure 3). The results indicated that Tspst is one component of the crude antigens but not from ES antigens.

### RT-PCR analysis

**Figure 4:**
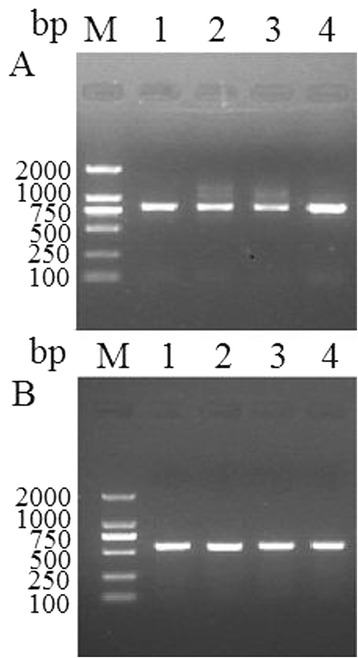
**RT-PCR analysis of Tspst gene transcript at** ***T. spiralis*** **different stages.** RT-PCR detection of mRNA transcription for the Tspst gene **(A)** and GAPDH gene **(B)** at *T. spiralis* different developmental stages. M: DNA marker; Lane 1: AW at 3 dpi; Lane 2: ML at 42 dpi; Lane 3: NBL; Lane 4: IIL at 2 hpi.

The transcript of the Tspst gene at different developmental stages of *T. spiralis* was determined using RT-PCR and the transcript of housekeeping gene GAPDH as a control. The mRNA transcript (807 bp) for the Tspst gene was detected at all the *T. spiralis* developmental stages (e.g., IIL at 2 hpi, AW at 3 dpi, NBL, and ML at 42 dpi) (Figure 4A). Furthermore, the primers for a standard gene (GAPDH) generated the expected size (570 bp) band in all of the samples (Figure 4B).

### Expression and immunolocalization of Tspst at different developmental stages

**Figure 5:**
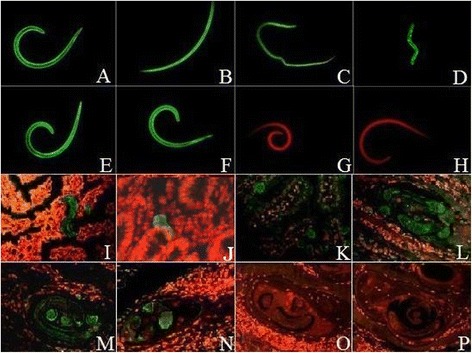
**Expression of Tspst at different developmental stages and immunolocalization in** ***T. spiralis.*** **A**–**H**: The results of IFT with whole parasite of *T. spiralis* different developmental stages reacted with anti-rTspst serum. The notable immunostaning is found in the entire bodies of IIL at 2 hpi **(A)**, AW at 3 dpi **(B)**, AW at 7 dpi **(C)**, NBL **(D)** and ML at 42 dpi **(E)**. The ML at 42 dpi reacted with infection serum **(F)** as a positive control; ML at 42 dpi did not show recognition by PBS **(G)** and normal serum **(H)** as a negative control. I–P: The results of IFT with the sections of intestines and skeletal muscles of infected mice reacted with anti-rTspst serum. The immunostaning is seen at the cuticle and internal organs of IIL at 2 hpi **(I)**, AW at 3 dpi **(J)**, AW at 7 dpi **(K)**, PEL at 19 dpi **(L)** and ML at 42 dpi **(M)**. The ML at 42 dpi reacted with infection serum **(N)** as a positive control; ML at 42 dpi did not show recognition by PBS **(O)** and normal serum **(P)** as a negative control.

The results of IFT with the whole parasite showed that the intense staining using anti-rTspst serum was found in the body of all the different developmental stages of *T. spiralis* (e.g., IIL at 2 hpi, AW at 3 dpi and 7 dpi, NBL, and ML at 42 dpi). When the sections of the intestines and skeletal muscle tissues of infected mice were incubated with the anti-rTspst serum, positive staining was found mainly at the cuticle and internal organs of IIL at 2 hpi, AW at 3 dpi and 7 dpi, pre-encapsulated larvae (PEL) at 19 dpi and ML at 42 dpi (Figure 5).

### Inhibition of larval invasion of IECs by anti-rTspst serum

When an IEC monolayer was overlaid with infective larvae suspended in a semisolid medium, the larvae invaded the monolayer and migrated through the cell monolayer. When anti-rTspst serum, serum from mice infected *T. spiralis* at 30 dpi and normal mouse serum were added to the medium, the invasion rate of the infective larvae in an IEC monolayer was 25.2%, 11.4%, and 79%, respectively (*χ*^2^ = 162.13, *P* < 0.05) (Table 1). The larval invasion rate in the anti-rTspst serum and the infection serum group were significantly lower than that in the normal serum group (χ_1_^2^ = 86.87, χ_2_^2^ = 139.67, *P* < 0.05).

**Table 1:** **Inhibition of anti-rTspst serum on the invasion of intestinal epithelial cells by** ***T. spiralis*** **infective larvae** ***in vitro***

**Type of sera**	**No. of larvae added**	**No. of larvae invaded**	**Invasion rate of larvae (%)**
Anti-rTspst serum	156.00 ± 7.07	39.50 ± 7.78	25.24 ± 3.84
Infection serum	154.60 ± 5.41	17.60 ± 3.29	11.36 ± 1.87
Normal serum	150.80 ± 6.30	118.80 ± 4.60	78.96 ± 5.75

### Immune protection of rTspst against challenge infection

**Figure 6:**
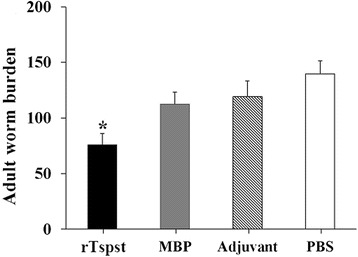
**The number of intestinal adult worms of vaccinated mice after being challenged with 300** ***T. spiralis*** **larvae.** Results are presented as the arithmetic mean of 10 mice per group ± standard deviation (SD). Asterisks (*) indicate statistically significant differences (*P* < 0.05) in worm burden compared to the three control groups.

Protective immunity against intestinal *T. spiralis* infection induced by the rTspst was observed in immunized BALB/c mice. After the challenge infection with *T. spiralis* ML, the mice immunized with the rTspst displayed a 45.7% reduction in their intestinal adult worms (Figure 6) compared with the groups vaccinated with PBS alone; further, the reduction of intestinal adult worms between the immunized and adjuvant or MBP tag groups was statistically significant (F = 44.097, *P* < 0.05).

## Discussion

In the present study, the Tspst gene encoding a 29 kDa protein from *T. spiralis* was successfully produced in an *E. coli* expression system, and the resulting rTspst protein and anti-rTspst serum were used to define some characteristics of the native 29 kDa protein of *T. spiralis*. After being purified, such recombinant protein has a good immunogenicity in mice and can be used as an immunogen to produce antibodies [32]. Our results showed that BALB/c mice immunized with the purified rTspst produced strong specific antibodies against the rTspst. Our Western blot analysis showed that the anti-rTspst serum obviously recognized the native Tspst protein with 29 kDa in the crude antigens of *T. spiralis* muscle larvae. The results of ELISA also indicated that the crude antigens were reacted with anti-rTspst serum, but the ES antigens were not recognized by anti-rTspst serum. The results demonstrated that the Tspst protein might be one of component of the somatic antigens of *T. spiralis* ML.

The characteristics of Tspst were identified at the gene transcription and protein expression levels using RT-PCR and IFT. As shown in Figure 4, the results of RT-PCR showed that the Tspst mRNA is transcribed during all the different developmental stages of *T. spiralis* (IIL, AW, NBL, and ML). The results of IFT revealed that the positive staining was widely found in the entire bodies of the whole parasites (IIL, AW, NBL, PEL, and ML) and sections of infected intestines and muscles incubated with the anti-rTspst serum. Proteasomes are the protein complexes inside all eukaryotes, archaea and in some bacteria. In eukaryotes, they are located in the nucleus and the cytoplasm [33]. Many cellular functions and processes are conserved in proteasomes including proteolytic functions, the regulation of cell cycle, apoptosis and transcription factor, enhancement of antigen presentation**.** The proteasomal degradation pathway is essential for many cellular processes, including responses to oxidative stress, the regulation of gene expression, and the cell cycle. It had been predicted that the proteasome subunit beta type-7 of *Caenorhabditis elegans* had a function in the ATP/ubiquitin-dependent non-lysosomal protein degradation [16]. The increased expression of a 19S proteasome subunit of *C. elegans* resulted in elevated somatic proteasome activity, clearance of damaged proteins and increased longevity [34]. The proteasomes have shown to play important roles in other parasites such as *Entamoeba* growth and encystation, *Leishmania mexicana* proliferation and *Trypanosoma cruzi* remodeling [35-37]. The previous studies showed that Tspst gene was an up-regulated genes in IIL compared to ML [14]. Our results showed that Tspst gene was transcribed and expressed during all the different developmental stages of *T. spiralis*, suggesting that the Tspst is an indispensable protein and plays an important role in the larval invasion and development of *T. spiralis* larvae.

Previous studies have shown that, when suspended in a semisolid medium and inoculated onto an IEC cell monolayer cultured *in vitro*, *T. spiralis* infective larvae invade and migrate through the cell monolayer, leaving serpentine trails of dead and damaged cells [13,25]. In this study, when anti-rTspst serum was added to the medium, the invasion rate (25.2%) of the infective larvae into the cell monolayer was significantly lower than the 79% of normal serum control groups, indicating that the anti-rTspst serum partially prevented the larval invasion of the IECs. This results demonstrated that antibodies against rTspst could reduce intestinal adult worms and protect IECs *in vitro* [38]. Anti-*Trichinella* antibodies could protect epithelia without the assistance of inflammatory cells, soluble cofactors, or mucus [39]. The mechanism of the blockage of the larval invasion of IECs by specific antibodies may be related to the formation of larval cephalic immune complexes that may physically block invasion or may interfere with sensory reception [40]. Furthermore, after the challenge infection with *T. spiralis* infective larvae, the mice immunized with the rTspst displayed a 47.5% reduction of adult worm burden in intestines. The results showed the rTspst induced a partial intestinal protective immunity in mice. Tspst might be a larval invasion-related protein, and could be considered as a potential vaccine candidate against intestinal *T. spiralis* infection.

## Conclusions

The present study showed that the Tspst gene was transcribed and expressed at all the different developmental stages of *T. spiralis.* Furthermore, Tspst was located mainly at the cuticle and internal organs of the parasite. The *in vitro* invasion assay showed that anti-rTspst serum partially prevented the larval invasion of IECs. The rTspst induced a partial protective immunity in the immunized mice, and could be considered as a potential vaccine candidate antigen against intestinal *T. spiralis* infection. Our results suggested that Tspst is an indispensable protein and plays an important role in the larval invasion and development of *T. spiralis* larvae, but its exact biological functions are needed to be further investigated.
